# Chicago Medical Society

**Published:** 1869-02

**Authors:** 


### CHICAGO MEDICAL SOCIETY.

FRIDAY EVENING, DEC. 18, 1868.

The meeting was called to order, Dr. Marguerat, President, in the chair.

Dr. Macdonald being absent, Dr. Gray was appointed Secretary, *pro tem.*

Dr. Holmes referred to a case under his care, of Gray's disease of the eyes—where they actually protruded from their sockets; owing to general relaxation of the recti muscles.

Dr. Marguerat asked what would be the prognosis in such a case?

Dr. H. replied, not favorable, as the man's father, sister, and brother were affected in the same way; the latter having lost one eye. The Doctor also spoke of the case of the young man whose lens dropped into the anterior chamber of the eye while stooping over, and which was reported at a previous meeting. He says that the patient returned home, and, six weeks after the operation, sat down in the evening and wrote a letter with remarkable ease and comfort, but the next morning found himself perfectly blind, owing to detachment of the retina. Dr. H. further remarked, that it was not uncommon for persons in good health to become blind from this cause.

Dr. Merriman asked if there was a way to correct this accident?

Dr. H. says not, if it is extensively detached.

The President then requested Dr. Bridge to open the discussion of the subject chosen at a previous meeting, *viz.:* "Would it be conducive to morality to adopt measures to prevent the spread of venereal disease"?

Dr. Bridge was of the opinion that any disease was injurious to morality, and thinks any measures adopted to prevent disease would improve morality; but is of the opinion any measures adopted to diminish the spread of venereal disease would increase prostitution; and thinks that many thousands now have nothing to do with prostitution, who would, were it not for fear of venereal disease. Read an extract from Dr. Andrews' investigations in Europe, who says there are 24 per cent. more prostitutes in cities where the license system is introduced.

Dr. Gray considered prostitutes a necessary evil, and did not see any better method than to license them, and when they become diseased send them to a free hospital for treatment. Says that in Paris there is *one* prostitute to 280, and in Chicago *one* to 230 of the inhabitants, and that 16 per cent. of all cases of disease in Chicago are venereal, and 16½ per cent. in Paris.

Dr. Wickersham considers this a very serious subject, and says that he has thought a great deal about it, as he has been called upon to treat a great many cases—as young men seek young physicians. Cited a case where $200 were offered a young lady on the Avenue, by a man who was afraid to go to a house of prostitution, for fear of *disease*, or being *pulled by the Police*. Hence making advances to virtuous girls. Thinks prostitution an unavoidable evil, and therefore favors the license system, believing it would be a protection to the virtuous, as well as prevent the spread of venereal disease. Says, were venereal disease only to affect the persons themselves, it would not matter so much; but it passes to the children, constituting congenital syphilis. Thinks the license system would also lessen this form of the disease.

Dr. Paoli is of the opinion that we can prevent this evil to a great extent by showing more Christianity, and through the influence of the churches, by preaching sermons appropriate for the prevention of evils in our society. Thinks if we had licensed houses, with careful examinations twice a week, that it would be productive of morality; also recommends that syphilitic as well as temperance apostles travel around the country, lecturing, as it would cause people to reflect, and consider the consequences of this disease and prostitution.

Dr. Merriman remarked, that, judging from statistics in this as well as other cities, he had become convinced that fear prevents the contraction of venereal disease only to a very limited extent. Thinks medical students not so much afraid of it as law students, although they have the opportunity of seeing it in the hospitals; but still they get it. If they do not go to houses of ill-fame, there will be more private women kept; hence believes, on the whole, it would be better to license them, and a certain portion of the city assigned to them; and, when diseased, that they be sent to hospital. By carrying out this system, he thinks that many men would be ashamed to go to that district, as people generally would judge that they went there for a special purpose. Consequently, thinks morality would be improved, and the spread of venereal disease diminished, by legalizing prostitution.

Dr. W. E. Clarke is of the opinion that if you make prostitution a crime, and punish it as such, that it will be productive of morality; but thinks there is no argument that can convince him that the licensing of houses of prostitution would produce such results.

Dr. Wickersham remarked, that those women living around in "Blocks" are the most dangerous and poisonous; as men have an opportunity of slipping up to their rooms unobserved. The Doctor favored the legalizing of regular institutions, and the arrest of every one who keeps a room; also, the arrest of every man who is found going to these *private* places. He says no man can go about this city but what will become convinced that prostitution is becoming alarmingly increased. He also mentioned the way our city ladies are brought up and educated, as being detrimental to morality, by being only fit to dress in silks and live on the Avenue. Says that young men living on a salary cannot afford to marry and support the style the young ladies have been accustomed to, but they can live singly and go in good society; hence, they refrain from marrying, knowing that the extravagance of their wives would inevitably reduce them to poverty.

Dr. Fisher says, that the less there is said about this subject the better; and that he does not know what we can do to prevent it. Thinks if all knew the effects of constitutional syphilis, that it would prove beneficial. Does not recommend the license system; but says we should do what we can to prevent it.

The Society then proceeded to miscellaneous business.

Dr. Bridge recommended, as the subject for the next discussion, "The Therapeutical Uses and Action of Mercury and its Salts," which was duly carried. Drs. Clarke, Merriman, and Loverin were appointed to open the discussion.

Dr. Hildreth stated that he was called at 5 P. M., one day last week, to see a young lady who had been poisoned by the escape of gas from a gas-stove in a bath-room. When the Doctor arrived, he found her unconscious, and respiration very slight. Immediately commenced artificial respiration, by compressing the ribs laterally; kept it up for ten minutes, when pretty good respiration was produced. The action of the heart was still feeble. Then gave the patient about a pint of strong hot coffee. In fifteen minutes, the circulation was good. The patient states that when she entered the bath-room she felt nauseated, which was the last she remembered.

The Doctor cited a similar case of poisoning by carbonic acid gas, as having occurred on shipboard; when he administered carbonate of ammonia and brandy without very beneficial results, but resorted to the coffee with entire satisfaction.

Society adjourned.

